# P24 Penicillin allergy de labelling: a whole system approach

**DOI:** 10.1093/jacamr/dlad143.028

**Published:** 2024-01-03

**Authors:** Heather Kennedy, Jo McEwen, Debbie Guthrie

**Affiliations:** NHS Tayside, Dundee, UK; NHS Tayside, Dundee, UK; NHS Tayside, Dundee, UK

## Abstract

**Background:**

Approximately 10% of patients have a documented allergy to penicillin on their health records.^1^ However, when checked up to 95% are thought to be incorrect^2^ with side effects such as nausea, vomiting and gastrointestinal upset often being documented as allergy. Inappropriate and over labelling of penicillin allergy is detrimental to individuals and healthcare systems as alternatives to penicillins are associated with more complications, adverse events, financial costs and extended length of stay. Unnecessary use of penicillin alternatives also increases the risk of antimicrobial resistance development. Penicillin allergy de-labelling is a viable option for patients within primary care and acute settings where they meet specific criteria, leading to financial benefits for the health board and optimal treatment outcomes for the patient. NHS Tayside is the first and only Board in Scotland to adopt a whole system approach to de-labelling patients in both acute and community settings. Through the implementation of evidence-based, collaborative and systematic risk assessment processes, clinical teams are able to identify patients who may be eligible for the penicillin allergy de-labelling programme.

**Methods:**

Within the acute sector, two pilot wards were identified. A Short Life Working Group (SLWG) was developed to oversee the de-labelling process and governance. Patients were identified by clinical teams and consent gained prior to carrying out the process. A single dose of oral flucloxacillin or amoxicillin was administered to the patient. The patient was monitored for any reaction as per Scottish Antimicrobial Prescribing Group (SAPG) algorithm.^3^ If the patient experienced no reaction, they were de-labelled, a request was sent to GP to remove the label and their details were added to a database held by the SLWG. A similar project was piloted in primary care across three Angus GP practices. The aim of the project is to identify a patient population that are labelled penicillin allergic but have received a subsequent prescription for a penicillin allowing a primary care SLWG to carry out a patient consultation with the aim of retrospectively de-labelling and updating the record. This will allow patients to receive optimal treatment options in the future.

**Results:**

Within the acute setting, the pilot was initiated in May 2022. Since then, 44 patients have been recruited to the penicillin allergy de-labelling process with 98% having no immediate reaction and 67% of labels removed from patient care records at the 3 month follow-up stage. Within primary care across three GP practices, 163 patients were eligible for the pilot. A total of 85 patients (52%) were contacted via telephone and a consultation on allergy status carried out. Overall, 42 patients (50%) were successfully de-labelled and patient records were updated.

**Conclusions:**

Following similar processes, there is potential for practitioners within the acute and community sectors to adopt this pilot as part of an integrated work plan across all wards and GP practices. By de-labelling patients within the community or acute sector, the patients’ records will be accurate and future treatment options can be optimized to ensure positive outcomes and minimize harm.
